# Silver nanoparticles have lethal and sublethal adverse effects on development and longevity by inducing ROS-mediated stress responses

**DOI:** 10.1038/s41598-018-20728-z

**Published:** 2018-02-05

**Authors:** Bin-Hsu Mao, Zi-Yu Chen, Ying-Jang Wang, Shian-Jang Yan

**Affiliations:** 1 Department of Environmental and Occupational Health, College of Medicine, National Cheng Kung University, No. 1, University Road, Tainan City, Taiwan; 2 Department of Physiology, College of Medicine, National Cheng Kung University, No. 1, University Road, Tainan City, Taiwan; 3 Department of Biomedical Informatics, Asia University, No. 500, Lioufeng Road, Wufeng District, Taichung City, Taiwan; 4 Department of Medical Research, China Medical University Hospital, China Medical University, No. 91, Hsueh-Shih Road, Taichung City, Taiwan

## Abstract

Silver nanoparticles (AgNPs) are widely used in the household, medical and industrial sectors due to their effective bactericidal activities and unique plasmonic properties. Despite the promising advantages, safety concerns have been raised over the usage of AgNPs because they pose potential hazards. However, the mechanistic basis behind AgNPs toxicity, particularly the sublethal effects at the organismal level, has remained unclear. In this study, we used a powerful *in vivo* platform *Drosophila melanogaster* to explore a wide spectrum of adverse effects exerted by dietary AgNPs at the organismal, cellular and molecular levels. Lethal doses of dietary AgNPs caused developmental delays and profound lethality in developing animals and young adults. In contrast, exposure to sublethal doses, while not deadly to developing animals, shortened the adult lifespan and compromised their tolerance to oxidative stress. Importantly, AgNPs mechanistically resulted in tissue-wide accumulation of reactive oxygen species (ROS) and activated the Nrf2-dependent antioxidant pathway, as demonstrated by an Nrf2 activity reporter *in vivo*. Finally, dietary AgNPs caused a variety of ROS-mediated stress responses, including apoptosis, DNA damage, and autophagy. Altogether, our study suggests that lethal and sublethal doses of AgNPs, have acute and chronic effects, respectively, on development and longevity by inducing ROS-mediated stress responses.

## Introduction

Nanotechnology—the creation, manipulation, and application of materials with nanoscale structures (1–100 nm)—has turned out to be a popular field of research in the 21^st^ century. Over the past few decades, advancements and breakthroughs in nanotechnology have revolutionized a variety of industrial sectors, such as medicine and electronics^1^. Nanofabricated silver particles (hereafter referred to as AgNPs) exhibit superior antimicrobial activity and unique plasmonic properties, and therefore, among the nanomaterials that are currently commercially available, AgNPs have received a relatively high amount of attention. Indeed, almost half of the registered nano-enabled consumer products (e.g., fabrics and textiles, bandages and wound dressings, detergents and cleaners, cosmetic creams and lotions, deodorants and antiperspirants, toothbrushes and toothpastes, tableware and cutlery, washing machines and refrigerators, and other household products) are said to contain AgNPs^2–4^. If not appropriately managed, such extensive and diverse usage of AgNPs would very likely lead to the release of these particles into the environment, which will inevitably be associated with high risks for ecosystems and/or humans^5,6^. To lay a solid scientific foundation for precautionary decision-making, it is essential for policymakers to scrupulously integrate nanotoxicological data from multiple sources into the policies they establish; these data include *in vitro*, *ex vivo* and *in vivo* examination or *in silico* computational modeling of potentially perilous effects of AgNPs and assessments of the risks associated with the frequent utilization of AgNPs^7^. Overall, most of the published research findings pertaining to mechanisms of AgNP-induced cytotoxic responses (increased production of reactive oxygen species (ROS), apoptosis, DNA damage, proinflammation, etc.) at the molecular and cellular levels were obtained via *in vitro* experiments^8^. In contrast, though a growing body of *in vivo* research has attempted to determine the toxic endpoints, such as LD50 (i.e. lethal dose, 50%) and phenotypically characterize the toxicity profiles (neurotoxicity, pulmonary toxicity, reproductive toxicity, etc.) of AgNPs^9–12^, the molecular and cellular mechanisms by which AgNPs exert adverse effects at the organismal level remain elusive.

The routes via which AgNPs gain entry into the body of an organism consist of oral ingestion, physical contact (through skin lesions or abrasions), pulmonary inhalation, and intravenous/intraperitoneal injection for either diagnostic or therapeutic purposes^13–17^. To date, only a few studies have investigated the possible toxic effects of orally administered AgNPs^18–23^. In 2009, the human dietary intake of silver, owing to the widespread use of silver compounds, was estimated at 70–90 μg.day^−1 ^^24^. However, this number likely underestimates the actual exposure levels; there has been a recent increase in the use of AgNPs in the food industry as components of the coatings of containers/processing machines or as packaging materials intended to prevent bacterial proliferation^25^. In addition, evidence from animal studies has shown that AgNPs are capable of being distributed to most organs following oral exposure^26,27^. The *in vivo* impact of dietary AgNPs has been explored in several mammals (weaned pigs, rats, mice, etc.); however, these investigations have led to inconsistent/controversial results^23,28–32^. For example, AgNPs contributed to a distinct dose-dependent increase in the body weight of pigs following a 14-day oral exposure but had no effect on the ileal mucosa of the pigs^28^. In mice and rats, some studies found that dietary AgNPs reduced the growth rate of the exposed mice and rats and affected the intestinal microvilli and the liver^22,29^, whereas other studies did not observe any effect of AgNPs on body weight^20,31^. In addition to the route of exposure, dosage and duration are also typically recognized as important determinants that affect the bioavailability, biodistribution and pathophenotypic outcomes of toxicant exposure^33^. Therefore, to elucidate the mechanisms underlying dietary-AgNP-induced toxicity, the effects of dosage and duration of exposure should be carefully considered.

Over the last decade, *Drosophila melanogaster* has been considered a powerful model system for toxicological studies (referred to as drosophotoxicology), including the assessment of nanotoxicity^34^. This model system is used not only because of its relatively short life cycle but also because of its ease of genetic manipulation. This organism possesses orthologs of approximately 65–80% of human genes. In addition, the use of this model species, which complies with the recommendations of the European Center for the Validation of Alternative Methods (ECVAM), raises only minor ethical objections^35^. As mentioned above, the detailed molecular and cellular basis of AgNP toxicity has yet to be explored, particularly at the organismal level. The purpose of this study was to explore the toxic effects and mechanisms of AgNPs at the organismal, cellular and molecular levels by using the *Drosophila* model system. Furthermore, to examine the effects of dosage and duration of exposure, the long-term impacts of dietary exposure to AgNPs were also evaluated in this study. Our results showed that lethal and sublethal doses of AgNPs have acute and chronic effects, respectively, on development and longevity by inducing ROS-mediated stress responses.

## Results

### Physicochemical characterization of synthetic citrate-capped AgNPs

**Figure 1:**
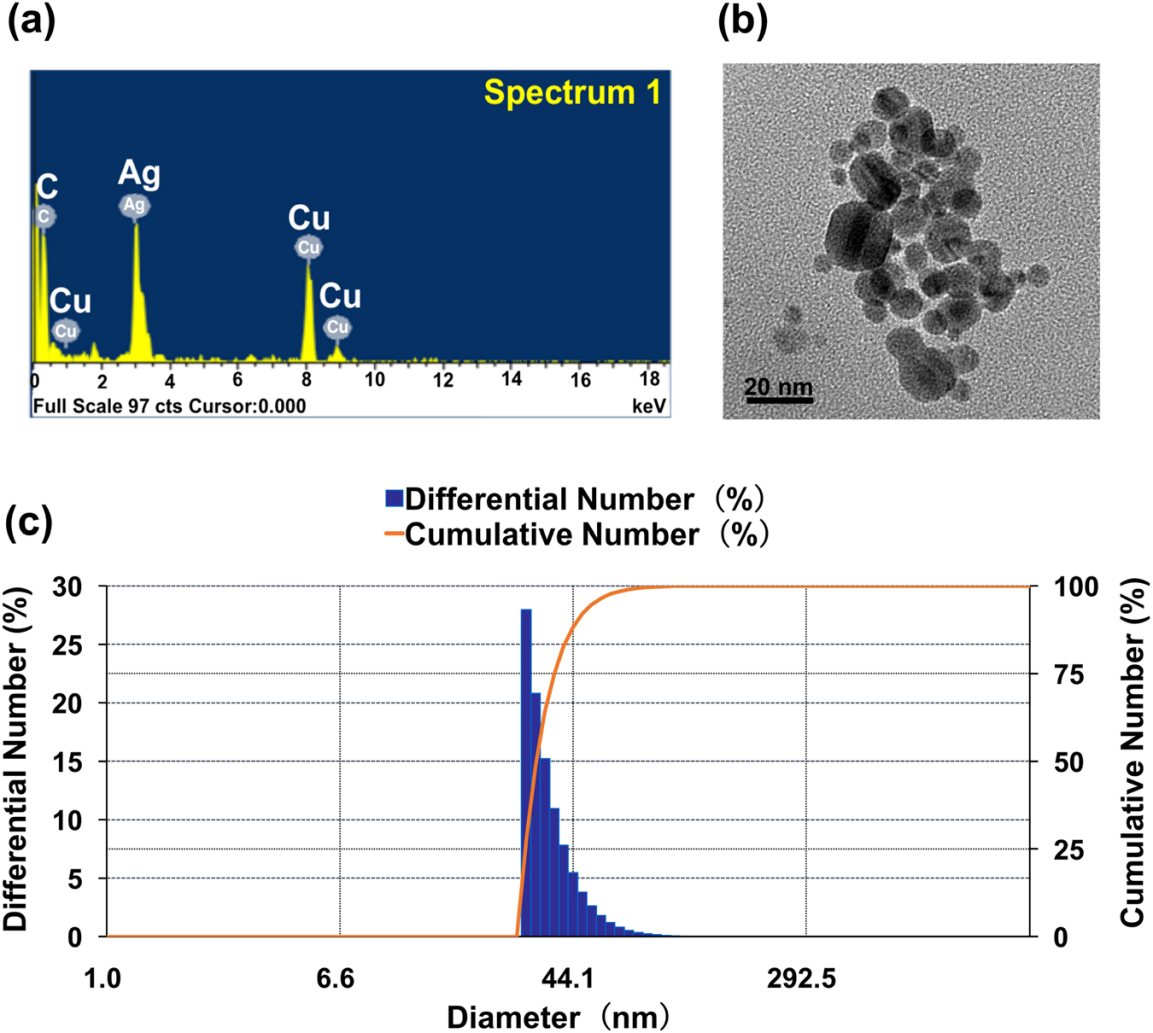
Chemical composition, morphology and size distribution of synthetic citrate-capped AgNPs. (**a**) Analysis of the elemental composition of the synthetic AgNPs by energy-dispersion X-ray spectrometry (EDX). (**b**) Shape and actual size of the synthetic AgNPs visualized under a transmission electron microscope (TEM). (**c**) Differential number-weighted particle-size distribution of the aqueous AgNP suspension measured using dynamic light scattering (DLS).

Prior to nanotoxicological assessment, physicochemical characterization of the nanoparticles being examined is required owing to the potential implications of physical and chemical properties in the manifestation of toxicity^36,37^. Physiochemical analyses were performed, following the standard methods, to demonstrate that the fabricated AgNP suspension was indeed eligible for this study. The EDX qualitative analysis validated that the primary particles of AgNPs are composed of nearly 100% elemental silver (the components of a TEM (transmission electron microscope) grid contributed to the detection of carbon and copper signal peaks) (Fig. 1a). As visualized under a transmission electron microscope (Fig. 1b), the synthetic citrate-capped AgNPs exhibited a spherical structure, with an actual mean size of 18.2 ± 8.7 nm (Table 1). The number-weighted size distribution data (Fig. 1c) indicates that the dimensions of these AgNPs in aqueous suspensions, though appearing rather uniform, are greater than the actual sizes of the particles; the average hydrodynamic diameter of the particles in these suspensions is 32.8 ± 4.4 nm (Table 1). Data pertaining to the other relevant physicochemical parameters of the examined AgNPs and the measurements of these parameters are summarized in Table 1.

**Table 1:** Physico-chemical properties of the synthetic AgNPs and their measurements.

Items of Physico-chemical properties	Measurement	Citrate-capped AgNPs
Morphology	TEM	Roughly spherical
Actual diameter (nm)	TEM	18.2 ± 8.2
Hydrodynamic diameter (nm)	DLS	32.8. ± 4.4
Polydispersity index (PDI)	DLS	0.304 ± 0.037
Chemical composition	EDX	Silver (100%)
Zeta potential (mV)	PALS	−23.6
Maximum absorbance (nm)	UV-Vis	391

### Lethal doses of AgNPs increase larval lethality and affect development

**Figure 2:**
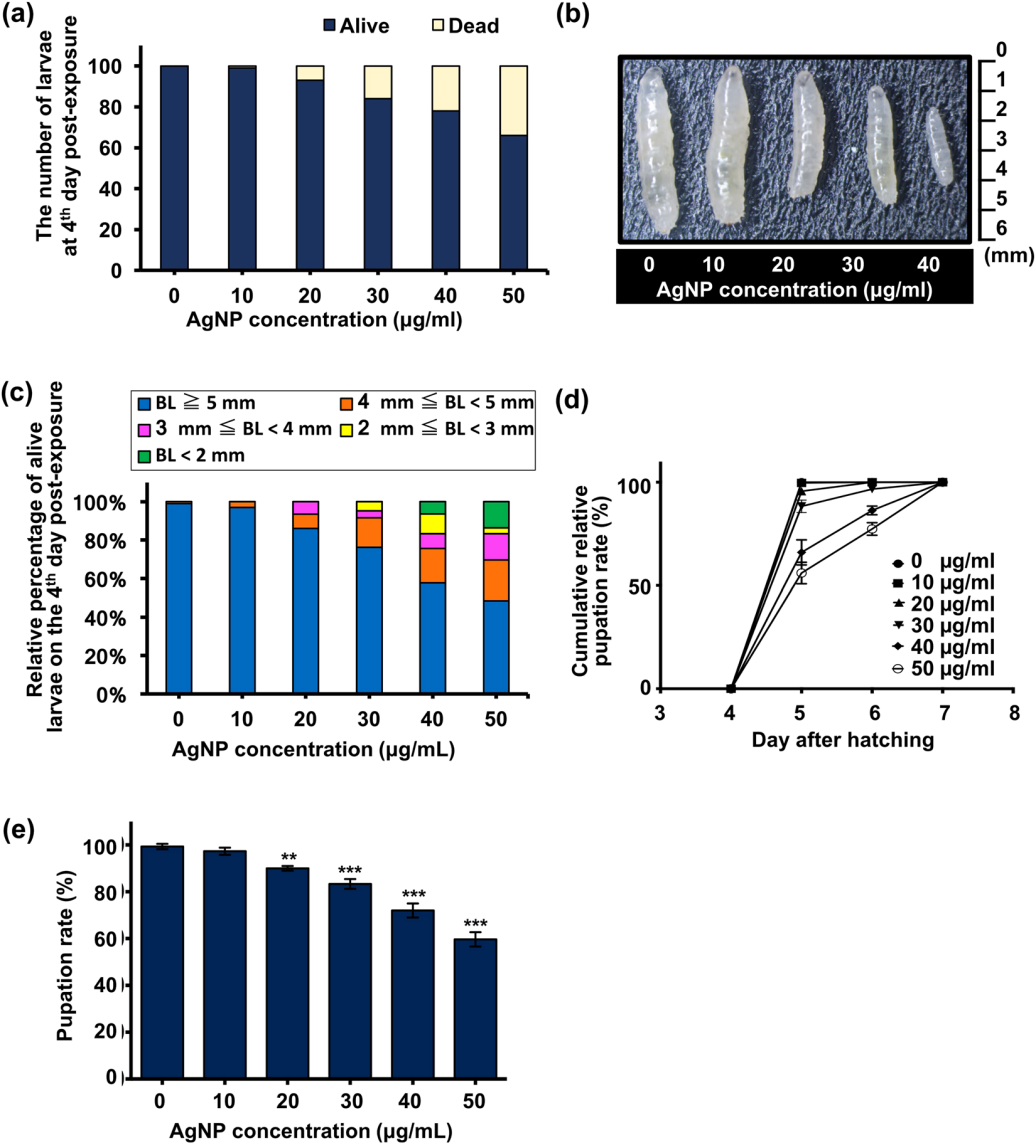
Effects of dietary AgNPs on developing larvae. (**a**) Stacked bar chart showing the relative number of viable and dead larvae on the 4^th^ day post-exposure to 0, 10, 20, 30, 40 and 50 μg/ml of AgNPs. Each group contained 100 larvae in total. (**b**) Stereomicroscopic image of the external morphological appearance of surviving larvae on the 4^th^ day post-exposure. (**c**) Stacked bar chart demonstrating the distribution of the body-length (BL) heterogeneity among the surviving larvae (i.e., relative percentage of viable larvae (derived from data shown in Fig. 2a) with respect to the range of body lengths) after 4 days of exposure. (**d**) Cumulative percentage of the surviving larvae (relative to the final pupal number) capable of undergoing pupation between the 4^th^ and 8^th^ day after hatching. (**e**) Percentage of surviving larvae that eventually turned into pupae (relative to the number of larvae in each group). For the experiments shown in Figs 2d and e, each vial was loaded with 100 larvae, and each group contained 300 larvae in total. **(*P* < 0.01) and ***(*P* < 0.001) denote significant differences between the control group and exposure groups.

Our study began with an investigation of the adverse effects of AgNPs at the organismal level. *D. melanogaster*, the model organism of choice for this study, was administered with conventional diets with or without citrate-capped-AgNP supplementation during the foraging (first, second, and early third instars) and wandering (late third instar) stages. We found a dose-dependent increase in mortality among larvae exposed to AgNPs on the 4^th^ day after exposure (Fig. 2a), the time point at which the untreated control larvae almost all molted into the third-instar stage. In the meantime, the level of body-length heterogeneity of the surviving larvae appeared to increase with increases in dosage (Fig. 2b and c). In addition to impaired growth, dietary AgNPs further contributed to a dose-dependent increase in the duration of the larval stage, leading to delayed onset of the pupation process (Fig. 2d). As shown in Fig. 2e, the percentage of larvae that survived the effects of the AgNPs and eventually entered the pupal stage decreased in a dose-dependent manner. Altogether, these results suggest that dietary AgNPs cause developmental toxicity in *Drosophila* larvae.

### Lethal doses of AgNPs protract pupal development and reduce eclosion success

**Figure 3:**
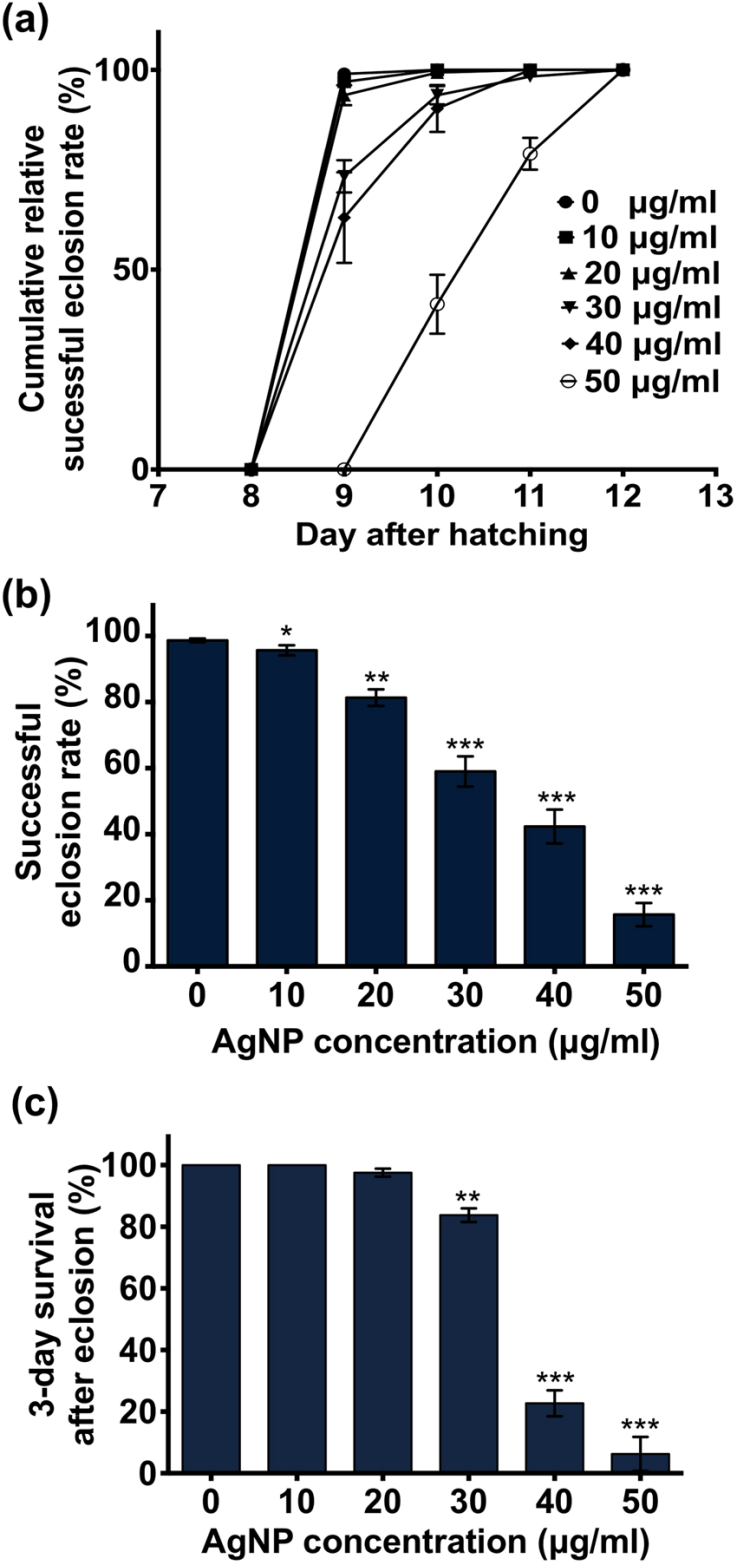
Effects of dietary AgNPs on the development of pupae and newly emerged adults. (**a**) Cumulative percentage of the flies eclosed daily (relative to the final eclosion number) between the 8^th^ and 12^th^ day after hatching. (**b**) Percentage of adult flies that successfully emerged from the pupae (relative to the number of larvae in each group). (**c**) Relative percentage of the successfully eclosed young adult flies that survived for 3 days after eclosion. For the experiments shown here, each vial was loaded with 100 larvae, and each group contained 300 larvae in total. *(*P* < 0.05), **(*P* < 0.01) and ***(*P* < 0.001) denote significant differences between the control group and exposure groups.

The pupal stage (i.e., the metamorphosis stage) generally lasts for 4 days (between the 6^th^ and 9^th^ day post-hatch), after which the adult fly emerges from the pupal case. Notably, even without exposure to AgNPs after pupation, AgNPs ingested during the larval stage continue to exert negative effects on flies during metamorphosis, leading to a dose-dependent prolongment of the pupal period (Fig. 3a). Exposure to higher doses of AgNPs (≥30 μg/ml) caused pupal death and unsuccessful eclosion of some emerging flies. Accordingly, the decrease in the successful eclosion of flies from the pupae occurred in response to increasing dosages of AgNPs (Fig. 3b). Even after successful eclosion, significant numbers of flies that received high doses of AgNPs died within three days (Fig. 3c). Taken together, these results indicate the cumulative toxicity potential of AgNPs on exposed organisms. Furthermore, consistent with the findings of a previous study^38^, we also found that ingestion of AgNPs during early larval development promoted demelanization (i.e., pigment whitening) of the adult cuticle; the higher the dose, the greater the level of whitening (Supplementary Fig. 1).

### Silver deposition occurs in response to both lethal and sublethal levels of AgNPs

**Figure 4:**
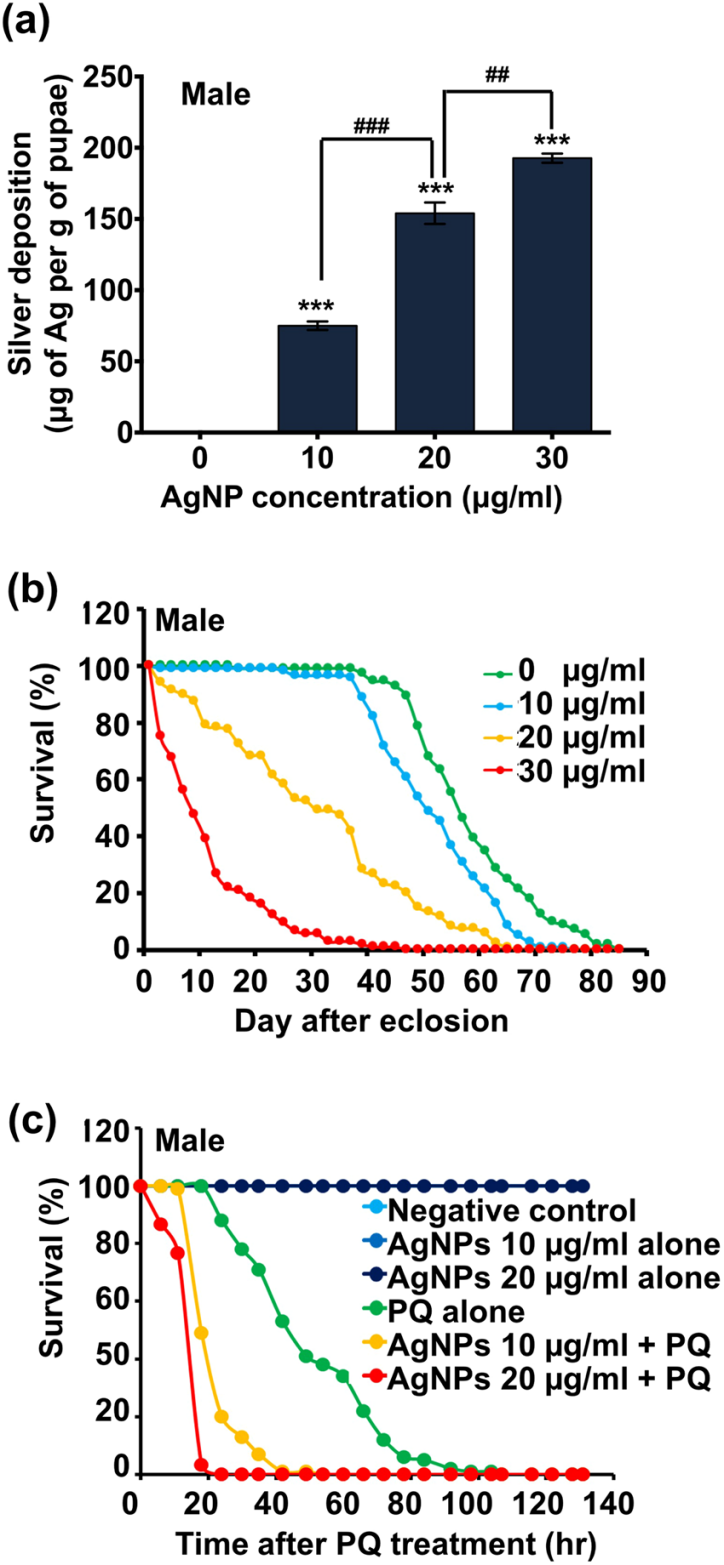
Cumulative effects of sublethal AgNP exposure on long-term survival and stress-resistance capacity. (**a**) Level of Ag deposition within the male pupae exposed to AgNPs (0, 10, and 30 μg/ml) during the larval stages. The pupae in each group, with a total weight of approximately 100 mg, were collected and then subjected to AAS analysis. (**b**) Long-term survival of the male adult flies that ingested sublethal or lethal doses of AgNPs during the larval stages. (**c**) Stress-resistance capacity of male adult flies that ingested sublethal doses of AgNPs during the larval stages; 20 mM paraquat (PQ) acted as a systemic stressor here. ^##^(*P* < 0.01) and ^###^(*P* < 0.001) denote significant differences between exposure groups; ***(*P* < 0.001) denotes significant differences between the control group and exposure groups.

A number of metallic nanoparticles are difficult for physiological clearance pathways to eliminate and may accumulate in certain organs of the body^26,39–41^. As previously shown in several rodent studies, following ingestion, AgNPs can be translocated through the intestinal barrier and then systemically distributed via the bloodstream or lymphatic system to secondary target organs (e.g., brain, liver, spleen, kidney and testes) where these particles cause functional and/or structural impairment^2,26,27,42–44^. The open circulatory system of *Drosophila*, similar to the mammalian cardiovascular and lymphatic systems, plays a role in facilitating wide tissue distribution of ingested substances following intestinal absorption. To determine whether the observed effects of dietary AgNPs on larval growth and development, on the duration of the larval and pupal stages, and on the success of adult eclosion might be correlated with the bioaccumulation of AgNPs, we performed an atomic absorption spectroscopic (AAS) analysis to quantitate the level of Ag deposition in the pupated flies that had been exposed to AgNPs. On the 3^rd^ day after pupation, the amount of Ag deposited within the AgNP-treated groups, in comparison with the untreated control, significantly increased as the dose of exposure increased (Fig. 4a and Supplementary Fig. 2a). These data suggest that ingested AgNPs are capable of accumulating in *Drosophila* tissues for a long time, even when the organisms are not under exposure.

### Sublethal AgNP exposure shortens the adult lifespan and compromises the stress tolerance capacity

As mentioned above, AgNPs appear to have the potential for long-term bioaccumulation within exposed flies, which introduces the possibility that accumulated AgNPs may exert chronic adverse effects on the lifespan and healthspan of *Drosophila*. To address this issue, we performed a longevity assay to assess whether dietary AgNPs decrease the lifespan of adult flies exposed to AgNPs during the larval stage. As shown in Fig. 4b and Supplementary Fig. 2b, AgNP exposure led to decreased lifespan, regardless of sex, in a dose-dependent manner. These results indicate that constant exposure of developing larvae to AgNPs, even at relatively low doses, can shorten the lifespan of the adult. As the healthspan of an organism is usually positively correlated with its lifespan, we investigated whether AgNP exposure also affects the health of adult flies. For this investigation, we carried out the paraquat challenge assay (which is a stress-resistance measurement that is usually an integral part of healthspan studies) to evaluate the stress tolerance capacity of adult flies that had been exposed to low doses of AgNPs (i.e., 10 and 20 μg/ml) during the larval stage. As shown in Fig. 4c and Supplementary Fig. 2c, the survival trends of these AgNP-treated adults were almost comparable to those of the untreated control group. Treatment with paraquat alone led to a significant decrease in survival compared to the untreated control. Intriguingly, pretreatment with low doses of AgNPs, regardless of sex, was shown to cause a sharp decline in the survival trends of flies challenged with equivalent amounts of paraquat in a dose-dependent manner. Thus, this result suggests that flies pre-exposed to AgNPs, even at low doses, are more prone to stress. Based on the above results, we concluded that dietary exposure to AgNPs during the larval stage not only interferes with larval growth, development and survival but also exerts long-lasting adverse effects on the lifespan and healthspan of adult flies following eclosion. In view of these conclusive findings, we aimed to further explore the toxicity mechanisms of AgNPs at the cellular and molecular levels.

### Lethal AgNP exposure leads to generation of ROS and activates the Nrf2-dependent response

**Figure 5:**
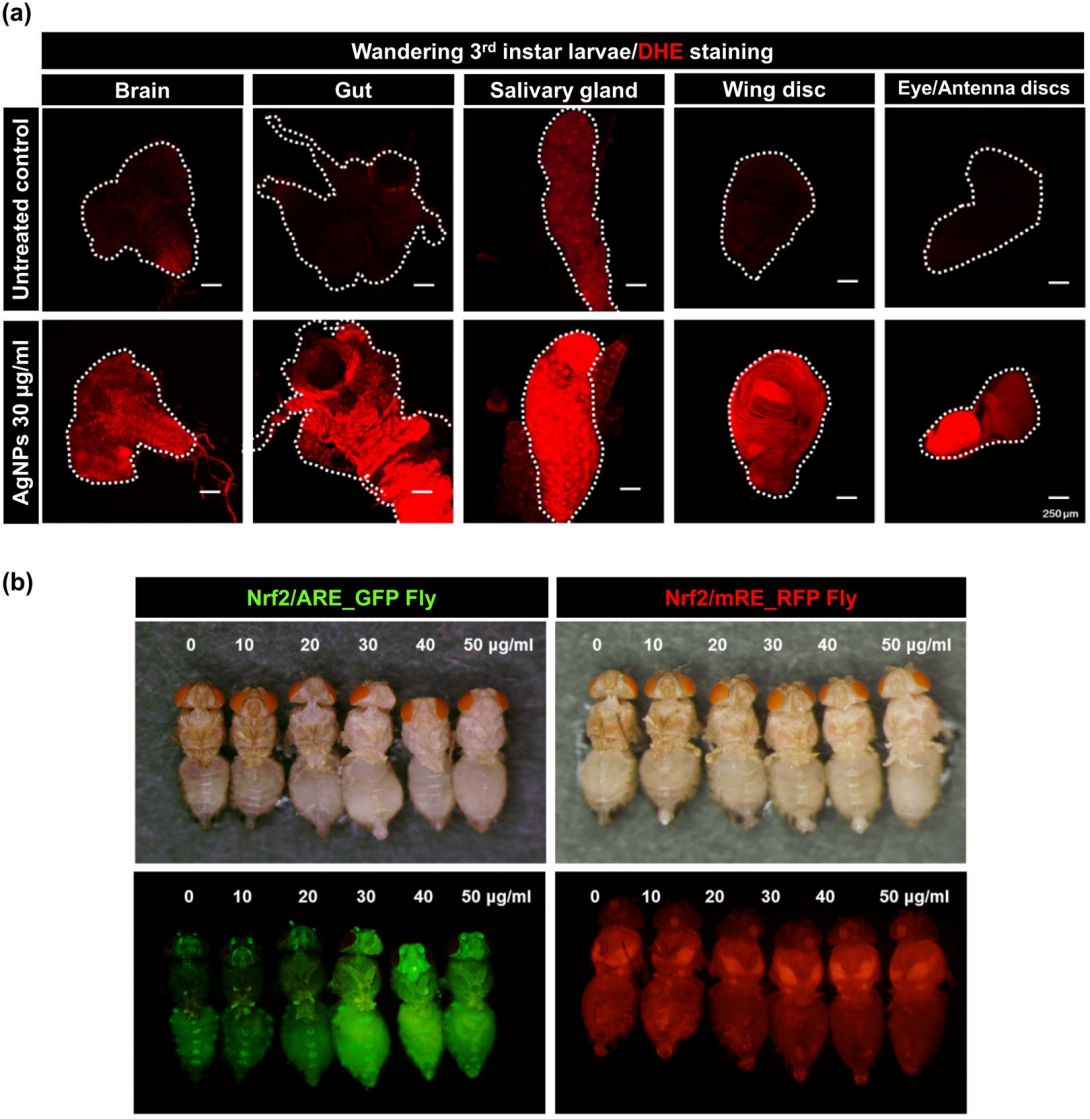
AgNP-induced generation of ROS and activation of the Nrf2-dependent antioxidant pathway. (**a**) The confocal microscopy image indicating systemic generation of superoxide (assessed by DHE staining) in multiple tissues (brain, gut, salivary gland, wing disc, and eye/antenna discs) of the wandering 3^rd^-instar larvae exposed to 30 μg/ml of AgNPs. (**b**) Dose-dependent enhancement of the GFP signal in adult Nrf2/ARE-reporter flies by dietary exposure to sublethal or lethal doses of AgNPs during larval stages [mRE-RFP flies were used as the unresponsive control].

Multiple lines of *in vitro* evidence have indicated that the generation of ROS is the mechanism underlying the cytotoxicity of AgNPs^8,45,46^. ROS act as an upstream regulator of various cellular responses, such as apoptosis, DNA damage and autophagy activation. In contrast, organismal effects of ROS generation in response to AgNP exposure remain elusive and have yet to be characterized on a system-wide scale. First, we determined the importance of ROS in dietary AgNP-induced *in vivo* adverse effects. As shown in Fig. 5a, dietary exposure to 30 μg/ml of AgNPs during the larval stage strongly elevated DHE fluorescence signals (indicative of superoxide generation) in a variety of *Drosophila* tissues (including gut, brain, salivary gland, wing disc, and eye/antenna discs) dissected from 3^rd^-instar wandering larvae, suggesting that systemic and massive ROS production resulting from dietary AgNP exposure account for the adverse effects detectable at the organismal level.

For the maintenance of cellular redox homeostasis, multiple antioxidant pathways might be activated in response to oxidative stress. Of these pathways, the Nrf2-ARE pathway is evolutionarily conserved in organisms from yeast to mammals. Massive ROS production can lead to nuclear translocation of Nrf2, which assists in the transcriptional regulation of a variety of antioxidant genes (e.g., *sod1*, *sod2*, *cat*, *gclc*, *gstD* and *gstE*) located downstream of the antioxidant response element (ARE)^47^. Therefore, we suggested that the Nrf2-dependent antioxidant pathway is activated in response to AgNP-induced ROS. To confirm this hypothesis, we subjected the Nrf2-ARE transgenic reporter fly line to AgNP exposure during the larval stage. As demonstrated in Fig. 5b, AgNPs elevated the levels of Nrf2 signaling activity (i.e., green fluorescence signal) in adult head and trunk segments in a dose-dependent manner. In contrast, such exposure had no effect on the transgenic flies harboring the mutant version of the antioxidant response element (mRE) (i.e., non-responsive control). In summary, these results imply that the Nrf2/ARE-dependent antioxidant system can be activated to attenuate AgNP-induced oxidative damage.

### Lethal AgNP exposure causes apoptosis and double-stranded DNA breaks

**Figure 6:**
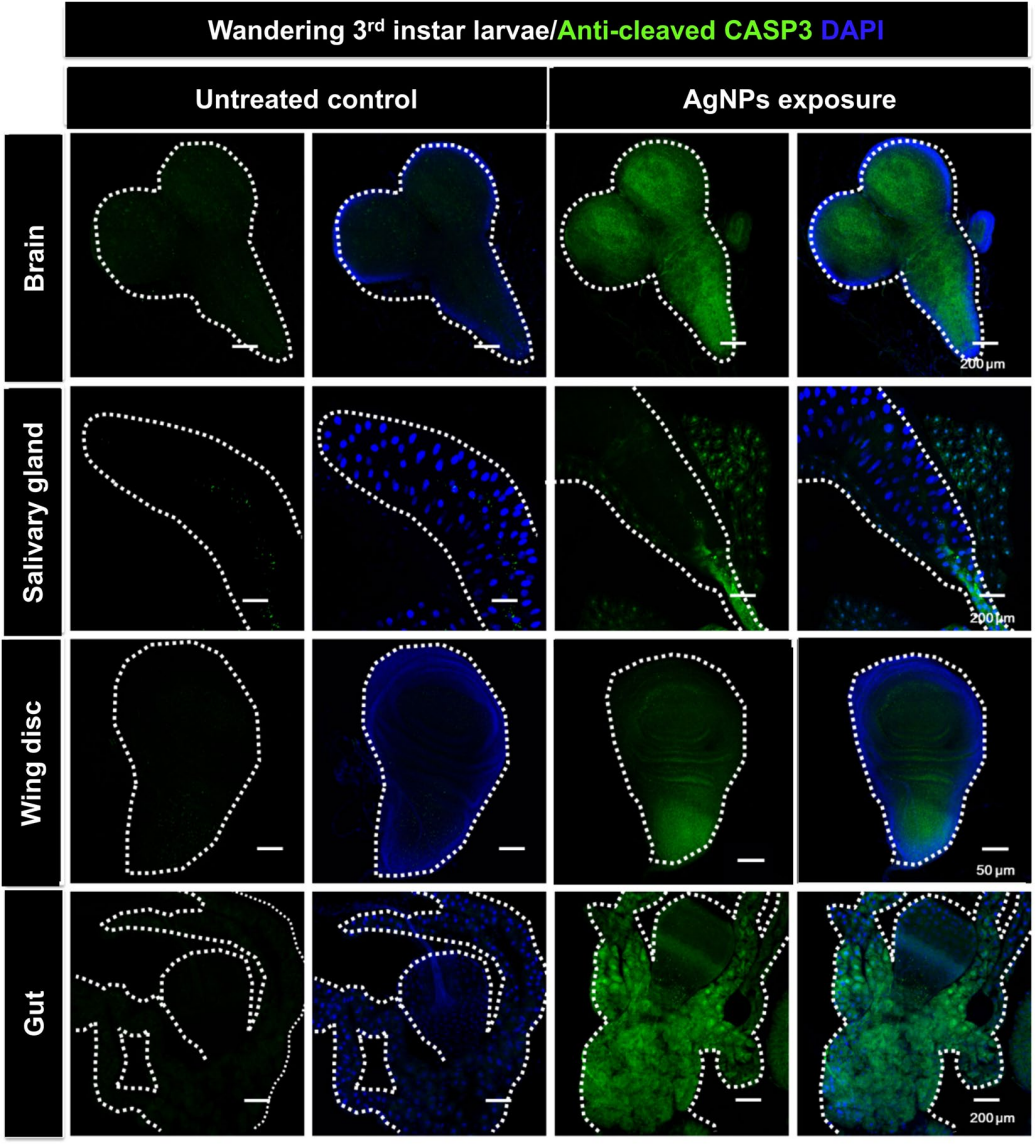
AgNP-induced occurrence of systemic apoptosis. Immunofluorescent signals of the apoptotic biomarker “active caspase 3” in multiple tissues (brain, salivary gland, wing disc and gut) dissected from AgNP-exposed 3^rd^-instar wandering larvae.

AgNP-induced ROS have been shown to contribute to the occurrence of apoptosis *in vitro*^48^. In this study, we performed immunohistofluorescence analysis, by detecting the fluorescence levels of the apoptotic marker “cleaved/active caspase 3” (the executioner of apoptosis), to evaluate whether AgNP exposure causes apoptosis *in vivo*. As shown in Fig. 6, dietary exposure to 50 µg/ml of AgNPs distinctly induced the activation of caspase 3 (i.e., green fluorescence signal) in the brain, salivary gland, wing disc and gut of the third-instar larvae.

**Figure 7:**
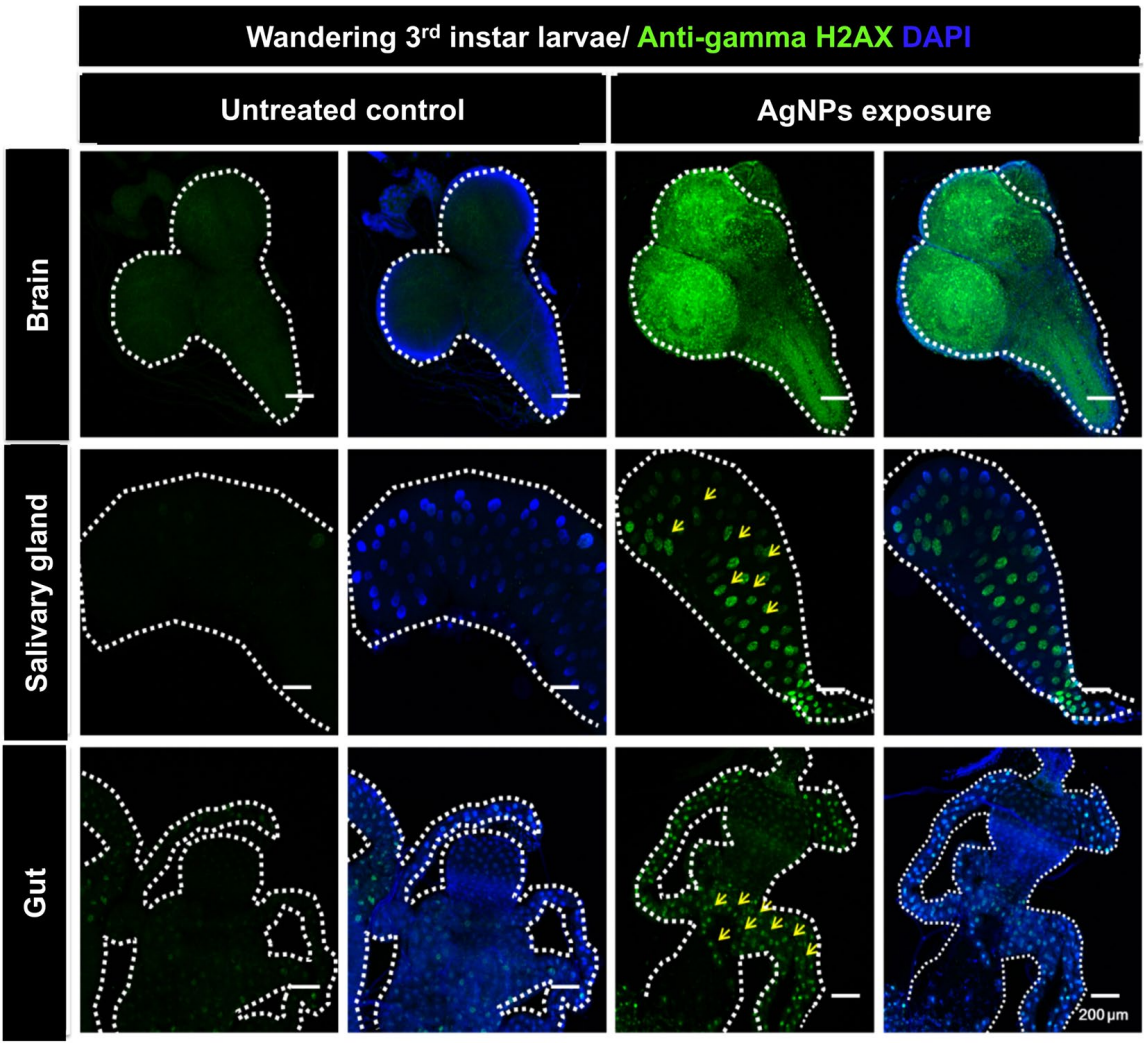
AgNP-induced occurrence of systemic DNA damage. Immunofluorescent signals of the double-stranded-DNA-break biomarker “γ-H2AX” in multiple tissues (brain, salivary gland, and gut) dissected from AgNP-exposed 3^rd^-instar wandering larvae.

Furthermore, massive ROS generation or impaired antioxidant response can lead to peroxidation of DNA, causing DNA strand breaks^49^. To investigate whether AgNPs possess genotoxic potential *in vivo*, we investigated the expression level of gamma-H2AX, a biomarker of double-stranded DNA breaks, in the nuclear foci of larval tissues. Based on the immunofluorescence images (Fig. 7), we determined that AgNPs cause DNA damage in larval brain, salivary gland, and gut, which suggests that AgNPs systemically cause DNA damage *in vivo* following ingestion.

### Lethal AgNP exposure leads to profound activation of autophagy

**Figure 8:**
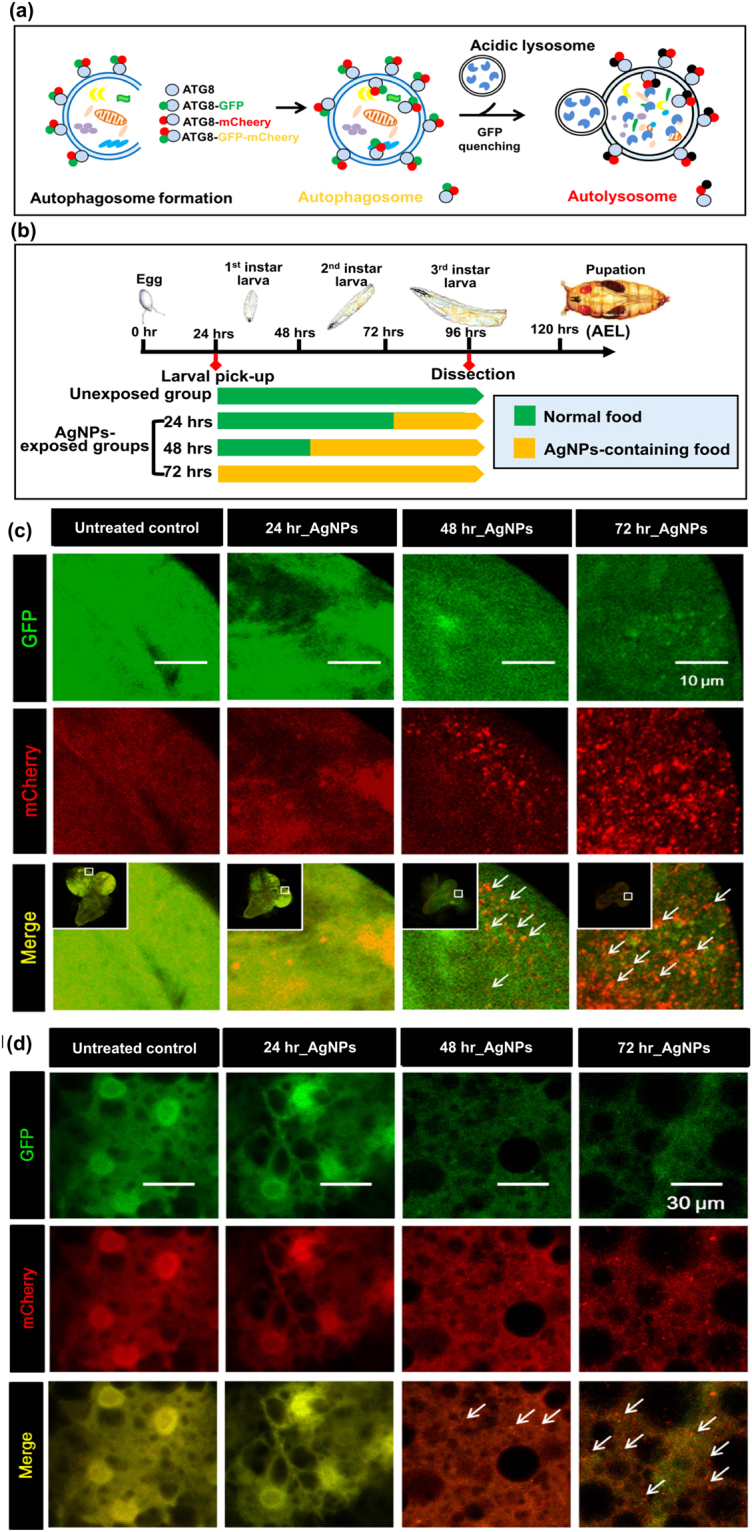
AgNP-induced autophagy activation. (**a**) The illustrative graph of the Atg8-GFP-mCherry reporter for qualitatively and quantitatively examining autophagic flux. (**b**) The experimental scheme for measuring AgNP-induced autophagy activation and subsequent progression. A time-dependent increase in the number of red fluorescent punctae (i.e., mCherry signals) were found in the (**c**) brain and (**d**) fat bodies of AgNP-exposed 3^rd^-instar wandering larvae.

A growing body of *in vitro* and *in vivo* evidence has noted the importance of autophagy in nanoparticle-induced toxicity. When activated at an appropriate stage and to a suitable extent, autophagy helps maintain cellular homeostasis^2^. However, overactivation of autophagy and/or subsequent disruption of autophagic flux has been observed in response to exposure to various metal nanoparticles (including AgNPs) *in vitro*^2^. In contrast, to date, there is insufficient *in vivo* evidence to address the role of autophagy in the systemic toxicity of AgNPs. To investigate this issue, we crossed a ubiquitous Gal4-driver fly line (daughterless Gal4) with a transgenic fly line carrying UAS-GFP- mCherry-tagged Atg8a to generate a reporter fly line that can be used to monitor the progression of autophagic flux *in vivo*. As illustrated in Fig. 8a, autophagosomes incorporated with fluorescently labeled Atg8a (the LC3 homolog in *Drosophila*) will be observed as punctae under a fluorescence microscope. The simultaneous presence of GFP (green fluorescence) and mCherry (red fluorescence) signals leads to the observation of yellow punctae, which represent autophagosomes. Upon fusion of autophagosomes with lysosomes, the acidic lysosomal environment causes quenching of GFP signals, resulting in the punctae merely emitting red fluorescence. Accordingly, red punctae represent autolysosomes. As the experimental scheme shows (Fig. 8b), we obtained the tissues from mid-third-instar AgNP-exposed larvae to evaluate whether ingested AgNPs trigger autophagy activation *in vivo*; this time-point was selected for the dissection because developmental autophagy occurs during the late-third-instar larval stage. To examine the time-dependent effects of AgNP exposure on the progression of autophagy, first-instar larvae were selected and dietarily exposed to 50 µg/ml of AgNPs for 24, 48 and 72 hrs. Interestingly, we found that AgNPs could enhance autophagy activation in a time-dependent manner in the larval brain (Fig. 8c) and in fat bodies (Fig. 8d). Our results suggest that sharp activation of autophagy in response to AgNP exposure is associated with adverse outcomes identified at the organismal level.

## Discussion

Due to the effective antimicrobial activity and unique plasmonic properties of AgNPs, these nanoparticles have been of particular interest for application in various sectors, such as commodity, healthcare, and electronic industries. The continued widespread usage of AgNPs will likely result in environmental contamination and human exposure, thus raising concerns pertaining to environmental safety and human health^2^. Though it has not yet been clinically noted that AgNP exposure has noticeable deleterious effects, a case report has shown that a woman acquired the condition of generalized argyria via the ingestion of colloidal AgNPs as an alternative health practice^50^. In addition, multiple lines of *in vivo* evidence have suggested that AgNPs can have acute adverse effects on various organ systems following oral, intraperitoneal, and intravenous administration^9,21,51^. Even though substantial progress has been made toward characterizing the acute toxicity of AgNPs at high doses (i.e., lethal doses), there remain large gaps in knowledge regarding the chronic and cumulative effects of sublethal levels of AgNPs on human health and safety. The present study suggests that in addition to increasing mortality and impairing larval development at lethal doses (≥30 μg/ml), exposure to sublethal doses (i.e., 10 or 20 μg/ml) of citrate-capped AgNPs can be biologically hazardous; based on the comparable dose-dependent patterns (Fig. 4a–c and Supplementary Fig. 2a–c), long-lasting bioaccumulation of the AgNPs ingested solely during the larval stage might account for the sustained and prolonged adverse impacts of AgNPs on pupal metamorphosis and on the longevity and healthspan (i.e., stress tolerance capacity) of adult flies. A recent study showed that even low bioavailability of dietary AgNPs, via chronic trophic transfer (i.e., food chain), has been found to lead to systemic toxic effects (inhibition of Na^+^/K^+^-ATPase and superoxide dismutase activity)^52^. More recently, Carew *et al*. have shown that chronic sublethal AgNP exposure disrupts thyroid hormone signaling during *Xenopus laevis* metamorphosis^53^. Together with these recent findings, our study suggests that more attention should be paid to the chronic cumulative effects of AgNPs on individual health and safety, even when exposed to sublethal or merely environmentally relevant concentrations of these nanoparticles.

The individual developmental stages of *Drosophila* are punctuated by molting and metamorphosis, and the events in each transition period are under stringent endocrine control^54^. Several vital hormones and neuropeptides take part in the regulation of *Drosophila* developmental transitions, among which the steroid hormone ecdysone (E) and the juvenile hormone (JH) are both considered master regulators of various biological processes, such as apoptosis, autophagy, and metamorphosis, necessary for successful development^55–58^. During the larval stage, ecdysone is chiefly produced in the prothoracic gland; after being released into the hemolymph, ecdysone is converted to an active form, 20-hydroxyecdysone (20E), by a microsomal cytochrome-P450-dependent monooxygenase that is expressed in many non-endocrine tissues^59^. Initiation of various gene expression cascades by the binding of 20E to its nuclear receptor underlies the molecular basis of physiological, morphological, and behavioral changes pertaining to molting and metamorphosis^60^. In contrast, JH exerts a “status quo” effect that prohibits the switching of the developmental program in 20E during larval molts and maintains the developmental arrest of imaginal disc primordia during the intermolt periods^61,62^. Though considered to have no apparent role in the onset of metamorphosis, JH prevents normal adult development when provided during pupation^63^. In the present study, we show that dietary exposure to AgNPs interferes with the growth and molting of developing larvae and with subsequent pupal metamorphosis (Figs 2c,d and 3a), suggesting that AgNPs potentially disrupt the synthesis of these hormones or inhibit their action.

The abovementioned hypothesis can be further explored by examining another piece of supporting data: the impairment of adult cuticular melanization resulting from AgNP exposure (initially demonstrated by Armstrong *et al*.^38^ and observed in our study, as shown in Supplementary Fig. 1). At a molecular level, the demelanization effect of AgNPs has been attributed to interference by AgNPs in intracellular copper homeostasis: the release of Ag^+^ from AgNPs blocks copper transporters, resulting in the sequestration of copper, which is a cofactor essential for activation of the tyrosinase involved in black melanin synthesis. In general, there are two types of color changes with respect to cuticles. Physiological color adjustments in response to environmental stimuli (e.g., background color, temperature, photoperiod, and population density) usually occur rapidly as a consequence of pigment migration under the control of neurohormones (known as chromatophorotropins)^64^. In comparison, morphological color changes, which are based on a slow and long-lasting process that involves variations in the levels of pigment and/or pigment cells, are mediated by the developmental hormones 20E and JH during developmental transition^65^. It has been noted that the absence of JH during larval molting prompts the epidermis to deposit melanin into the new pupal cuticle, which is followed by ommochrome (a visual pigment responsible for the red color of typical adult eyes) synthesis^66,67^. Similarly, in addition to melanin production, ecdysteroids, namely, ecdysone and its homologues, are also correlated with the initiation of ommochrome synthesis^68^. Intriguingly, even though AgNPs interrupt cuticular melanization, AgNP exposure has no influence on the eye color of adult flies (Supplementary Fig. 1). Detailed mechanisms and pathways associated with the impairment of cuticular melanization by AgNPs without affecting visual pigmentation have yet to be elucidated. With regard to the developmental toxicity of AgNPs, the whitening capacity of AgNPs toward adult cuticle also suggests the effects of AgNPs on the interaction between 20E and JH and on the synthesis and activation of these hormones.

Our study clearly indicates that dietary exposure to AgNPs induces tissue-wide enhancement of ROS generation in developing larvae (Fig. 5a), and a prolonged, dose-dependent activation of the Nrf2-dependent antioxidant pathway occurs in response to AgNPs, even at the pupal stage, during which there is no food intake (Fig. 5b). These results suggest that the Nrf2-dependent antioxidant system plays a crucial role in counteracting AgNP-induced ROS and consequently in defending against cytotoxicity *in vivo*. Previously, AgNP treatment has been shown to elevate the expression levels of several Nrf2 target genes, including enzymes participating in the biosynthesis of glutathione (GSH); however, the fold-increase values observed for the expression levels of these genes decreased significantly upon Nrf2 knockdown^69^. Consistent with this finding, the nuclear accumulation of Nrf2, which is inducible by AgNP treatment, was hampered by pretreatment with the potent antioxidant N-acetyl cysteine (NAC)^69^. Therefore, this study clearly shows the importance of ROS in AgNP-mediated Nrf2 activation. In 2016, Osborne *et al*., via biochemical investigation and gene expression analysis, verified the role of the Nrf2-Keap pathway in the regulation of various genes involved in oxidative detoxification upon exposure to AgNPs^70^. With emerging technologies for genetic manipulation in *Drosophila*, we alternatively chose a rather sensitive *in vivo* reporter assay to directly visualize the activation level of the Nrf2 signaling pathway in living *Drosophila* under a fluorescence stereomicroscope. It is worth noting that the Nrf2-ARE antioxidant system, being likely to cooperate with other alternative homeostatic pathways, can be activated in a dose-dependent manner to alleviate AgNP-induced oxidative damage. However, if the total level of damage by an extremely lethal dose of AgNPs exceeds the capacity of the individual to tolerate or repair, the exposure will ultimately lead to the demise of the organism due to multiple insuppressible cytotoxic responses.

In addition, we also provide *in vivo* evidence that dietary AgNPs activate a series of ROS-mediated cytotoxic pathways (e.g., apoptosis, DNA damage and autophagy), which have been previously examined in multiple *in vitro* studies^8,48,71,72^. While almost consistent with the findings of a previous *in vivo* study using *Drosophila*^73^, our confocal microscopic images of the whole-mount double-fluorescence-labeled dissected larval tissues provide direct information regarding the molecular and cellular basis of AgNP-induced tissue-wide adverse effects (i.e., developmental toxicity and increased mortality) at the organismal level. Mechanistically, we show that ingestion of AgNPs during the larval stage leads to caspase-3-dependent apoptosis and double-stranded DNA breaks in multiple larval tissues. However, it cannot be concluded that such cytotoxic responses are directly attributable to AgNPs because several lines of research have suggested that the ROS generated and the dissolved silver ions may contribute to the tissue-wide occurrence of apoptosis and DNA damage^74–78^.

A number of studies have shown that the autophagic pathway is activated in response to numerous nanoscale particles; although, in certain cases, whether this activation is attributable to the chosen nanoparticles themselves remains disputable^79^. Our recent work first revealed that AgNPs can induce autophagy *in vitro*^80^. In the present study, we demonstrate *in vivo* that dietary exposure to AgNPs can systematically activate autophagy. It is well known that autophagy plays an essential role in maintaining cellular homeostasis. Autophagy is, in general, activated in response to cellular stress (such as ROS and specific organellar injury) to remove misfolded protein aggregates and damaged organelles^81^. Unfortunately, this cellular defense system may be overactivated under extreme or chronic stress, which could compromise cell viability^82^. Our study demonstrates that AgNPs induce high levels of ROS tissue-wide. Furthermore, the brain and fat body of the mid-third-instar larvae, having been exposed to a lethal dose of AgNPs, also exhibit significant time-dependent increase in the number of autolysosomes, suggesting that dietary AgNPs aggravate autophagy activation over time. Taken together, these results suggest an association between AgNP-induced ROS and autophagy activation. In other words, the excessive ROS accumulation induced by AgNPs may contribute to substantial enhancement of autophagy activation.

On the other hand, a growing body of *in vitro* research has suggested that AgNPs also act as potent autophagic flux inhibitors since they mostly gain entry into normal cells via the endo-lysosomal route and possibly contribute to lysosomal impairment (alkalinization and/or rupture)^2,83^. Consequently, autophagosome-lysosome fusion (i.e., the progression of autophagic flux) would be inhibited due to the internalization of AgNPs via endocytosis, and the resultant inhibition of autophagic flux may consequently aggravate the cytotoxic effects (e.g., DNA damage, apoptosis, and mitochondrial impairment) exerted by AgNPs^2^. In this study, we did not conclusively observe that dietary AgNPs induce defective autophagic flux in the larval brain and fat bodies; our data showed that AgNP-activated autophagy mostly proceeded to the final stage of autolysosome maturation (Fig. 8c and d). We propose that the systemic propagation of dietary AgNPs to target tissues and ensuing internalization by the constituent cells are key processes that determine the occurrence of defective autophagy flux *in vivo*. Whether dietary AgNPs potentially act as autophagic flux disrupters following systemic dissemination and how this phenomenon interferes with the physiological functioning of the exposed animals require further study.

## Materials and Methods

### *Drosophila* strains

All fly stocks were kept under 65% humidity and 25 °C with a 12-hour light cycle. Wild type flies (derived from cross of *w*^*1118*^ and *Oregon R* strains) were used for larval lethality/adult survival assay, DHE staining assay, anti-cleaved caspase 3/anti-γ-H2AX immunohistofluorescence assays, longevity assay, and paraquat challenge assay. Nrf2 (nuclear factor erythroid 2-related factor 2)/ARE (antioxidant response element)-GFP (green fluorescence protein) reporter flies and mRE (mutant version of the antioxidant response element)-RFP (red fluorescence protein) non-responsive controls flies were gifts from Dr. Dirk Bohmann at University of Rochester, NY, USA. Both daughterless Gal4 driver flies (BL55849) and UAS-Atg8a-GFP-mCherry reporter flies (BL37749) were purchased from the Bloomington *Drosophila* Stock Center.

### Fabrication of citrate-coated AgNPs

The method for synthesis of 20 nm citrate-coated AgNP was modified by a formerly described protocol^84^. Briefly, silver nitrate (1 mM) and sodium citrate (3.2 mM) pyogen-free aqueous mixture was drop-wisely and slowly titrated with 20 mL of 100 mM cold sodium borohydride solution at 4 °C under 800 rpm stirring. Following this process, the resultant colloidal AgNPs suspension was stored at 4 °C and kept from light.

### Dietary AgNP exposure

The standard cornmeal/sucrose/yeast/agar medium was the major ingredient of the diet fed to *Drosophila* larvae of the control and exposure groups^85^. For the exposure groups, 20-nm citrate-coated-AgNP solution was subjected to ultrasonication (800 W) at 4 °C for 10 min and then well mixed with the standard *Drosophila* medium (maintaining the temperature at approximately 60 °C) to prepare AgNP-containing diets of different final concentrations (10, 20, 30, 40, and 50 µg/ml). To perform exposure experiments, 1^st^-instar larvae (2–6 hrs after hatching) were selected and fed normal or AgNP-containing diets. Unless otherwise stated, the exposure period started from the 1^st^ larval stage to the late 3^rd^-instar larval stage (when the larvae stop feeding).

### Atomic absorption spectrometry

To quantify the level of silver deposition within *Drosophila* following dietary exposure to AgNPs (10, 20, 30 μg/ml) during the larval stage, atomic absorption spectrometry (AAS) was used to examine the cumulative amount of silver in pupal larvae (at 3-day post-pupation) that weighed 100 mg in total. The collected pupae of experimental and untreated groups were respectively treated with 1.5 mL of nitric acid (69%) for 4 hrs at 50 °C, followed by another 4 hrs at 100 °C. Once fully digested, the samples were diluted with 2% nitric acid to an acceptable concentration (approximately 200 to 300-fold dilutions depending on the exposure doses) and then subjected to analysis using the graphite furnace atomic absorption spectrometer (Perkin Elmer AAnalyst^TM^ 600). Triplicate measurements of the absorption for the sample were taken for each exposure dose.

### Longevity assay

For long-term adult lifespan analysis, dietary exposure to AgNPs was carried out over all the larval stages. After eclosion, 3-day-old flies, which presumably had mated, were separated by sex and further divided into four groups (n = 120 flies each, 10 flies per vial). The surviving flies were transferred into vials containing fresh standard food every 2 or 3 days. Every 2 days, the number of surviving flies was recorded until all the flies had died.

### Paraquat challenge assay

The stress resistance capacity of the 3-day-old flies (dietarily exposed to AgNPs during the larval stages) was evaluated by the paraquat challenge assay described previously^86^. Briefly, the newly emerged adult flies were separated by sex and divided into 6 groups (n = 120 flies each, 10 flies per vial) and starved for 3 hrs before the paraquat treatment. After starvation, the vials containing filter papers were soaked with 1 ml of 20 mM paraquat solution (using 5% sucrose stock solution as the solvent). Every 6 hours, the number of dead flies was recorded until all the flies had died.

### Dihydroethidium (DHE) staining

The level of AgNPs-induced ROS production (superoxide anions in this study) in *Drosophila* larval tissues was determined by dihydroethidium (DHE) staining described previously^87^. Briefly, the dissected larval tissues were incubated in Schneider’s *Drosophila* medium (GIBCO, cat.no. 11720) supplemented with 30 µM DHE for 5 minutes in the dark. After incubation, tissues were washed 3 times with PBS and then immediately mounted on slides with Schneider’s *Drosophila* medium. Images were photographed by a Zeiss confocal microscope.

### Nrf2/ARE reporter gene induction assay

Nrf2-responsive ARE-GFP reporter flies and non-responsive mRE-RFP controls (gifts from Dr. Dirk Bohmann at University of Rochester)^88^ were used to evaluate the level of AgNPs-induced oxidative stress *in vivo*. First instar larvae were collected and subjected to control and AgNPs-containing diets (N = 100 per group), and the exposure was kept until they turned into the late-third instar larvae. After eclosion, fluorescent images of the adult flies were taken with the Olympus stereo fluorescence microscope.

### Whole-mount immunofluorescence analyses of apoptosis and DNA damage

The completely inverted larvae were fixed in 4% paraformaldehyde for 10 minutes and then washed 4 times for 10 minutes with 0.3% PBST. After washing, the inverted larvae were incubated with the primary antibodies (indicated below) at 4 °C overnight. The rabbit anti-cleaved caspase 3 (1:250, Cell Signaling Technology, MA, USA) and rabbit anti-phosphorylated H2AX (1:500, Rockland, PA, USA) primary antibodies were used to quantitate the levels of AgNP-induced apoptosis and DNA damage (double-stranded breaks), respectively. After overnight incubation, the inverted larvae were washed 3 times with 0.3% PBST and then incubated with goat anti-rabbit 488 secondary antibodies (1:300, Molecular Probes) for 2 hours. Finally, tissues obtained from further dissection of the inverted larvae were mounted on a clear glass slide with DAPI-containing VECTASHIELD Antifade Mounting Medium (Burlingame, CA, USA). Images were captured with the Zeiss LSM780 microscope.

### Statistical analysis

All experiments were conducted in triplicate. Statistical analyses were carried out using the Student’s *t*-test. *P* values < 0.05 were considered statistically significant.

### Data Availability

The datasets generated during and/or analysed during the current study are available from the corresponding author on reasonable request.

## Electronic supplementary material

Supplementary figures
