# Supplementary material for: Silver nanoparticles have lethal and sublethal adverse effects on development and longevity by inducing ROS-mediated stress responses
Source: Sci Rep. 2018 Feb 5;8:2445. doi: 10.1038/s41598-018-20728-z (PMC5799281; doi:10.1038/s41598-018-20728-z)
Supplement: Supplementary file 1 — Supplementary figures [file 41598_2018_20728_MOESM1_ESM.pdf]

Silver nanoparticles cause lethal and sublethal adverse effects on development and longevity by inducing ROS-mediated stress responses

Bin-Hsu Mao[1, 2, #], Zi-Yu Chen[1, 2, #], Ying-Jang Wang[1, 3, 4*], Shian-Jang Yan[2**]

1. Department of Environmental and Occupational Health, College of Medicine, National Cheng Kung University, No.1, University Road, Tainan City 701, Taiwan (R.O.C.)
2. Department of Physiology, College of Medicine, National Cheng Kung University, No.1, University Road, Tainan City 701, Taiwan ROC
3. Department of Biomedical Informatics, Asia University, No. 500, Lioufeng Road, Wufeng District, Taichung City 41354, Taiwan (R.O.C.)
4. Department of Medical Research, China Medical University Hospital, China Medical University, No. 91, Hsueh-Shih Road, Taichung City 40402, Taiwan (R.O.C.).

Bin-Hsu Mao (S78011039@mail.ncku.edu.tw);

Zi-Yu Chen (S78051021@mail.ncku.edu.tw);

Ying-Jan Wang (yjwang@mail.ncku.edu.tw);

Shian-Jang Yan (johnyan@mail.ncku.edu.tw)

*Correspondence: Ying-Jan Wang, Department of Environmental and Occupational Health, College of Medicine, National Cheng Kung University, 1 University Road, Tainan City 701, Taiwan (R.O.C.). Tel: +886 6 235 3535 ext. 5804. E-mail address: yjwang@mail.ncku.edu.tw

** Correspondence: Shian-Jang Yan, Department of Physiology, College of Medicine, National Cheng Kung University, 1 University Road, Tainan City 701, Taiwan (R.O.C.). Tel: +886 6 2353535 ext. 5437. E-mail: johnyan@mail.ncku.edu.tw

#: equal contribution

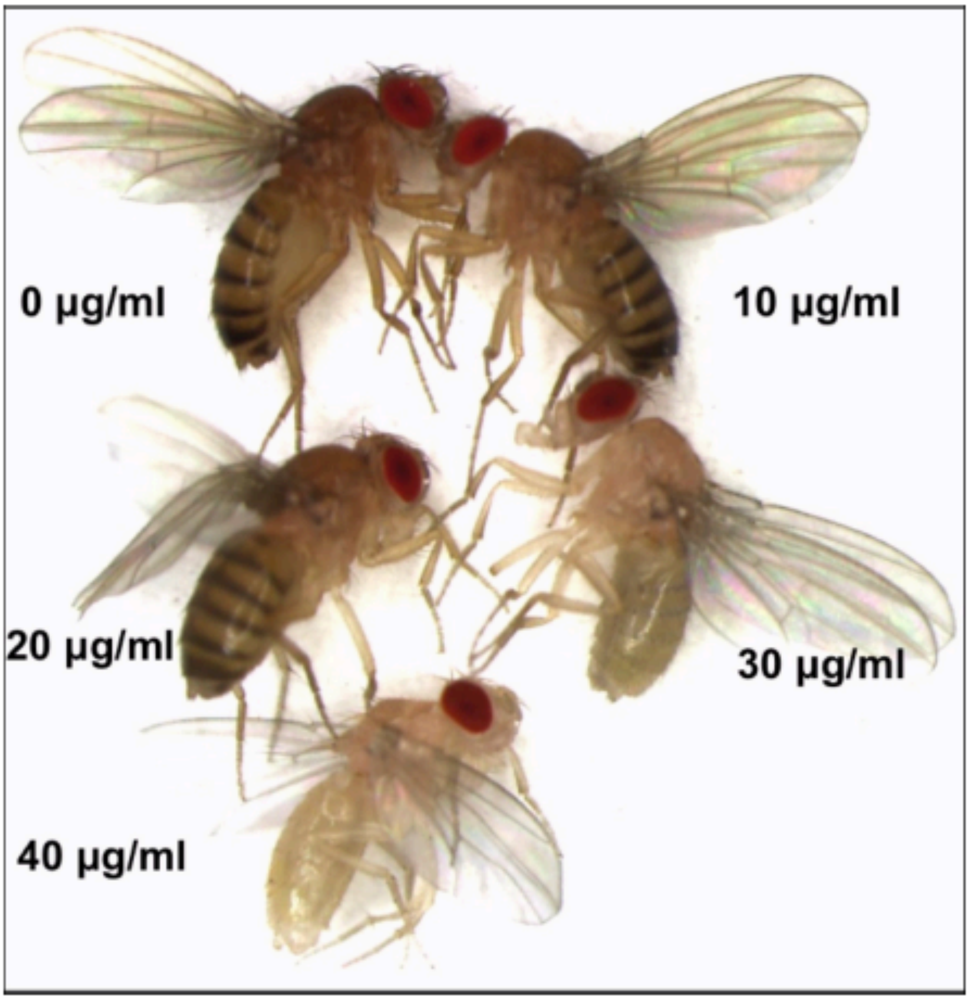

Supplementary Figure 1. AgNPs dietary exposure during the larval stages leads to cuticular demelanization of adult flies.

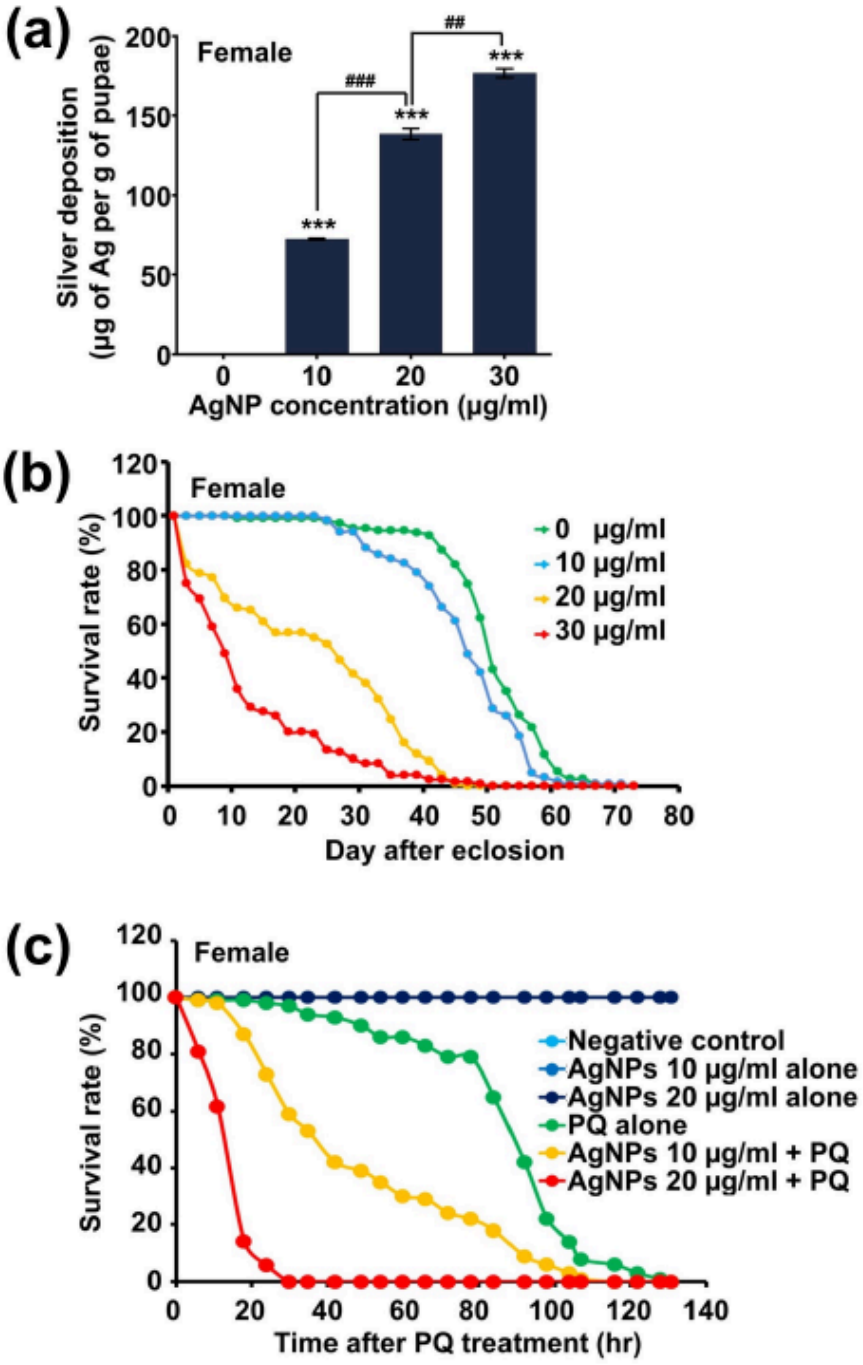

Supplementary Figure 2. Accumulation of ingested AgNPs during the larval stages impairs the long-term survival and stress-resistance capacity of female adult flies. (a) Level of Ag deposition within the male pupae having been exposed to AgNPs (0, 10, 30, 50 µg/ml) during the larval stages. (b) Long-term survival of the female adult flies having been exposed to AgNPs during the larval stages. (c) Stress resistance capacity

of female adult flies having ingested low-dose AgNPs during the larval stages. ## ($P<0.01$) and ### ($P<0.001$) denote significant differences between exposure groups; *** ($P<0.001$) denotes significant differences between control group and exposure groups.
